# Stab-wound in Abdomen—Laparotomy and Recovery

**Published:** 1891-09

**Authors:** R. Menger

**Affiliations:** San Antonio


For Daniel's Texas Medical Journal.

### STAB-WOUND IN BLADDER.—LAPAROTOMY.—RECOVERY.

BY R. MENGER, M. D., SAN ANTONIO.

STAB-WOUND of the bladder being of rare occurrence, the following case is herewith put on record:

Hilario Abrijo, a young intelligent Mexican, aged 22 years, and another party hailing from the land of the Montezumas, had an old quarrel to fight out and on Sunday night, toward morning, August 16, while Abrigo and a friend were coming from a fandango-theatre, he met his antagonist, who, without much warning, plunged a good sized dagger into the lower abdomen of Abrijo, penetrating his bladder. He reeled and fell into the arms of his friend who had him removed to his home. About two hours after this I was summoned to him and found him greatly prostrated and complaining bitterly of his bladder, having tenesmus, and unable to void any urine. On examining the abdomen, there was a sharp wound, about ¾ inch long, very near and to the left of the median line and just above the symphisis pubis. His bladder was undoubtedly filled to the brim at the time of stabbing. On probing the probe entered obliquely and deep down to the pelvic cavity toward the left abdominal side, but the integuments were that much, so to say, "displaced," that it was impossible to enter the bladder with a probe. There was very little oozing of bloody urine from the probed wound, and on introducing a catheter into the bladder through the urethra only a small quantity of deep colored bloody urine, intermixed with blood coagula could be removed. As the bleeding and the emaciation of the patient continued, I concluded to make laparotomy in order to drain the bladder thoroughly and check the bleeding. Drs. Braunnagel, Berry and Oldham kindly assisted in the operation, which was done at the city hospital immediately after the wounded man was removed there. The incision, about three inches long, was made in the linea alba, in the rear of the stab-wound. In the depth of the incision, near the bladder, a lacerated place was found from which a continuous bleeding was kept up; it was sewed up with a few catgut stitches and the bleeding stopped, but there was still some hemorrhage further below, coming from the lacerated bladder direct. The patient was now laid on the left side, the wound kept open and the extravasated urine was drained off as much as possible and afterward washed out with borated water. As it was not possible to get to the stab-wound opening in the bladder direct, the same being seated extra-peritoneal and very deep—the bladder in a collapsed condition—we concluded to insert a draining tube into the opening of the bladder, but it was so difficult to find the opening that only after introducing a catheter per urethram high up in the bladder, toward the direction of the stab-wound, and lifting the walls of the bladder with the catheter, it was possible, after considerable fruitless efforts before, to find the stab-wound opening of the bladder, through which then a drainage-tube was pushed over the mouthpiece of the catheter (still in the bladder), the same retracted and pulling the drain-tube in the bladder, where it was kept in situ by a wire stitch through the tube and abdominal walls. The bladder now was washed out through this drain-tube, after inserting an elastic catheter through the urethra. Considerable bloody urine and coagula came away but gradually only the clear drainwater came away from the bladder and no bloody urine appeared any more afterward. The patient was a great deal relieved after a few hours, and he passed a quite comfortable night.

The temperature was, next morning, 100½%; on the next following morning, 101%, and after that nearly entirely normal. A double ice-bag was constantly kept upon both sides of the drain-tube for several days, after covering the wound and next surroundings with layers of plain absorbent cotton. The drain-tube had been inserted too deep in the bladder the first day and its mouth seemed to lodge against the wall of the bladder, and therefore would not work well, but after lifting the tube a little it drained well and only clear water came away. From the third day on the patient could urinate freely from the urethra, but the drain-tube was left two days longer. On the fifth day already the fellow sat up out of bed in a chair and continued to improve until recovered.

P. S.—I have seen the patient at the hospital this morning; he is up and about. The opening in the bladder is entirely closed, at least there has been no oozing after the seventh day of injury, and the laparotomy wound is healing fast.

